# Identification of biomarkers associated with pediatric asthma using machine learning algorithms: A review

**DOI:** 10.1097/MD.0000000000036070

**Published:** 2023-11-24

**Authors:** Kexin Lin, Yijie Wang, Yongjun Li, Youpeng Wang

**Affiliations:** a Heilongjiang University of Chinese Medicine, Harbin, Heilongjiang Province, China; b The Second Affiliated Hospital of Heilongjiang University of Chinese Medicine, Harbin, Heilongjiang Province, China.

**Keywords:** biomarker, machine learning, pediatric asthma, WGCNA

## Abstract

Pediatric asthma is a complex disease with a multifactorial etiology. The identification of biomarkers associated with pediatric asthma can provide insights into the pathogenesis of the disease and aid in the development of novel diagnostic and therapeutic strategies. This study aimed to identify potential biomarkers for pediatric asthma using Weighted Gene Co-expression Network Analysis (WGCNA) and machine learning algorithms. We obtained gene expression data from publicly available databases and performed WGCNA to identify gene co-expression modules associated with pediatric asthma. We then used machine learning algorithms, including random forest, lasso regression algorithm, and support vector machine-recursive feature elimination, to classify asthma cases and controls based on the identified gene modules. We also performed functional enrichment analyses to investigate the biological functions of the identified genes.We detected 24,544 genes exhibiting differential expression between controlled and uncontrolled genes from the GSE135192 dataset. In the combined WCGNA analysis, a total of 104 co-expression genes were screened, both controlled and uncontrolled. After screening, 11 hub genes were identified. They were AK2, PDK4, PER3, GZMH, NUMBL, NRL, SCO2, CREBZF, LARP1B, RXFP1, and VDAC3P1. The areas under their receiver operating characteristic curve were above 0.78. Our study identified potential biomarkers for pediatric asthma using WGCNA and machine learning algorithms. Our findings suggest that 11 hub genes could be used as novel diagnostic markers and treatment targets for pediatric asthma. These findings provide new insights into the pathogenesis of pediatric asthma and may aid in the development of novel diagnostic and therapeutic strategies.

## 1. Introduction

Asthma is a chronic inflammatory disease of the airways characterized by recurrent airflow obstruction resulting from edema, bronchospasm, and increased mucus production.^[1]^ Childhood asthma is a major global health problem that not only affects children’s physical health but also has a profound impact on their psychological and social development.^[2]^

Asthma is the most prevalent chronic disease in children and is diagnosed in 1 in 12 children under 18 years of age in the United States.^[3]^ The disease is marked by its heterogeneity and is characterized by fluctuating and reversible airway obstruction, along with airway hypermobility. Children diagnosed with asthma have annual healthcare expenditures that vary between $3279 and $13,612.^[4]^ This figure is significantly higher than in children without asthma. Increases in healthcare costs have been associated with emergency department visits and hospitalizations resulting from inadequate management of asthma and asthma attacks. It is estimated that half of children under 18 years of age have poorly managed asthma, with at least 42.7% reporting at least 1 asthma exacerbation per year.^[5]^

In asthmatic children, 3 major inflammatory phenotypes have been recognized: eosinophilic, neutrophilic, and oligomelanocytic. Most prevalent is eosinophilic asthma, characterized by airway inflammation, blood eosinophils exceeding 2% to 3%, elevated IgE levels, and elevated fractional exhaled NO levels.^[6]^ Although there are some differences in the prescribing criteria for administrative approval between the US Food and Drug Administration and the European Medicines Agency, currently available biologic therapies, such as omalizumab, mepolizumab, benralizumab, dupilumab, and 1ezepelumab, are widely recognized for the treatment of variant asthma in children.^[7]^

The aim of this study was to find key genes associated with childhood asthma, screen diagnostic biomarkers more comprehensively, efficiently, and accurately, and provide a basis for understanding the mechanism of asthma. This may have significant implications for the diagnosis and treatment of asthma in children.

## 2. Data and methods

### 2.1. Design and methods

**Figure 1.**
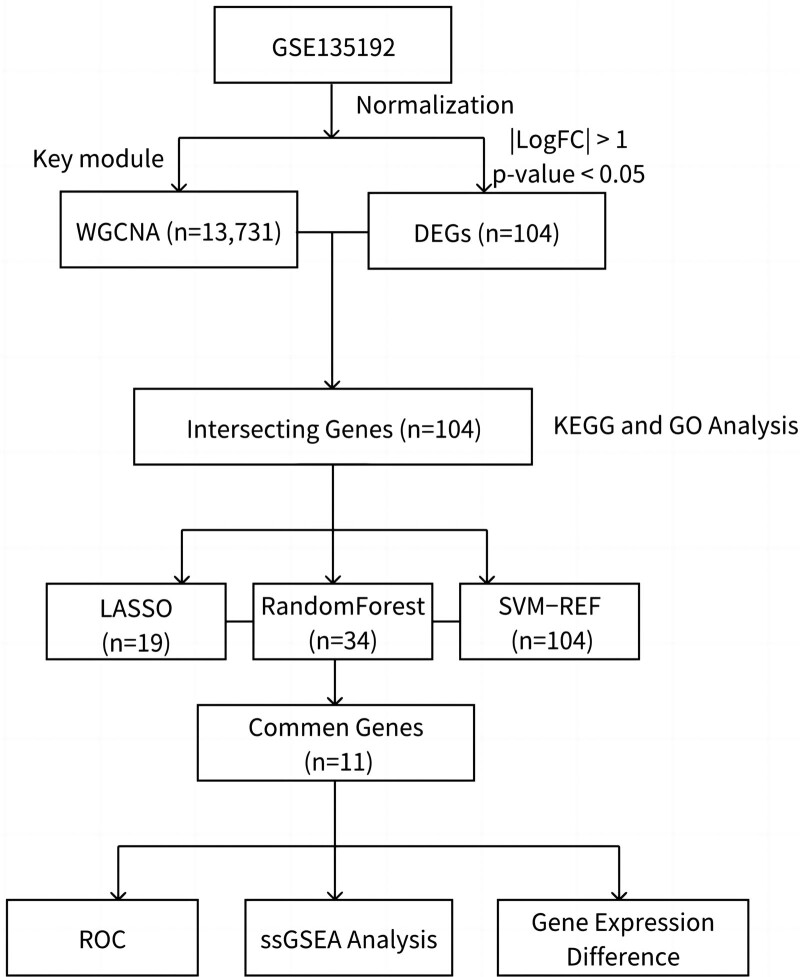
Flowchart.

Three machine learning algorithms were used to cross-confirm and analyze gene expression profiles in asthmatic children based on whole transcriptome sequencing. The intersection results of differential genes from multi-platform databases were selected and further extracted to find key genes associated with childhood asthma. Afterwards, enrichment analysis and validation were performed for these potential biomarkers. Figure 1 illustrates the workflow chart of data preparation, processing, analysis, and validation.

### 2.2. Data acquisition

Common morbidity complexity and common genetic correlations of pediatric asthma were investigated using bioinformatics as well as systems biology approaches from microarray and RNA-Seq datasets from the NCBI database GEO (https://www.ncbi.nlm.nih.gov/geo). A raw human gene expression dataset for pediatric asthma was collected. The GEO accession number dataset is GSE135192, and the platform is GPL16791 (Illumina HiSeq 2500).

### 2.3. Identification of differentially expressed gene (DEGs)

To identify DEGs in the dataset, R software (v.4.3.1) was used to screen the DEGs in the serum samples of asthmatic and non-asthmatic children. Due to the small sample size of this study. Based on small sample data with random variance model correction, a T-test was used to screen DEGs. Genes with a screening-adjusted *P* ≤ .05 and log_2_ FC ≥ 1 (fold change = 2) were identified as DEGs.

### 2.4. Weighted gene co-expression network analysis (WGCNA) analysis

Key modules were picked according to the correlation between the module members and the significance of genes. We used the WGCNA package in R software to screen hub genes. WGCNA is a useful technique for discovering clusters of genes that exhibit high levels of correlation. These clusters, known as modules, can then be summarized using the module eigengene or an intramodular hub gene. WGCNA also allows for the exploration of relationships between different modules and external sample traits through the use of the eigengene network methodology. Furthermore, WGCNA can calculate module membership measures. Correlation networks provided by WGCNA enable the application of network-based gene screening methods, which can aid in the identification of potential biomarkers or therapeutic targets.^[8]^ First, the correlations among the genes were calculated, and a topological overlap matrix (TOM) was constructed. The diss TOM between the genes was calculated using the following formula: diss TOM = 1 − TOM. A phylogenetic clustering tree was then established based on the hierarchical clustering of dissTOM; that is, genes with similar expression were divided into the same modules. Genes from key modules were used for subsequent analysis.

### 2.5. Enrichment analysis

Kyoto encyclopedia of genes and genomes (KEGG) and gene ontology (GO) enrichment analyses were performed on the DEGs using the R software.

### 2.6. Hub genes

The genes between DEGs and genes in the key modules via WGCNA were identified to obtain candidate genes. Then, we implemented Random Forest, lasso regression algorithm (LASSO), and SVM-RFE in R, respectively, to further identify hub genes, which were the genes identified by Random Forest, LASSO, and SVM-RFE. Different approaches have their own advantages and disadvantages. LASSO is easy to interpret and train. It also has a good benchmark. However, it cannot learn complex feature relationships. Overfits with a large number of features. SVM-RFE can perform both linear and nonlinear classification and regression. Its drawback is that scaling to large datasets is often difficult. As for Random Forest, its advantage lies in learning how important each feature is to the prediction. Individual decision trees are human-readable, allowing interpretation of how a decision is made. The drawbacks are that they are less appropriate for regression, and many decision trees are hard to build.^[9]^

### 2.7. Hub genes verification

Hub genes were verified by evaluating the diagnostic efficacy using receiver operating characteristic (ROC) curves and appraising the expression profile in the data. Firstly, we plotted ROC curves using ROC packages and determined the area under ROC curves. An area under curve (AUC) value > 0.6 signified that the data was a good fit for the gene, and a *P* value < .05 indicated that the value was of statistical significance. The validated genes were identified as robust diagnostic biomarkers for pediatric asthma.

### 2.8. Single sample gene set enrichment analysis (ssGSEA) of hub genes

To assess the distribution of immune cell subtypes in each sample of the GSE135192 dataset, we employed the GSVA function in the R package. This function utilizes the ssGSEA algorithm, which deconvolutes gene expression profiles and provides quantitative fractions of immune cells within a single sample.

## 3. Results

### 3.1. Screening of DEGs

**Figure 2.**
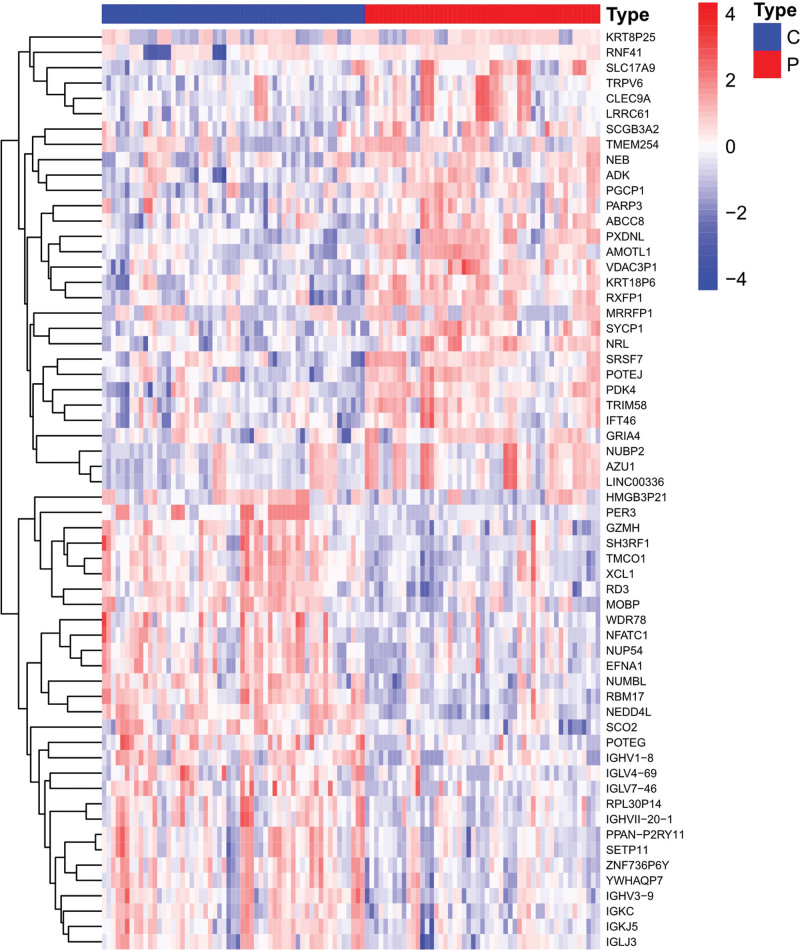
DEGs heatmap. DEG = differentially expressed gene.

**Figure 3.**
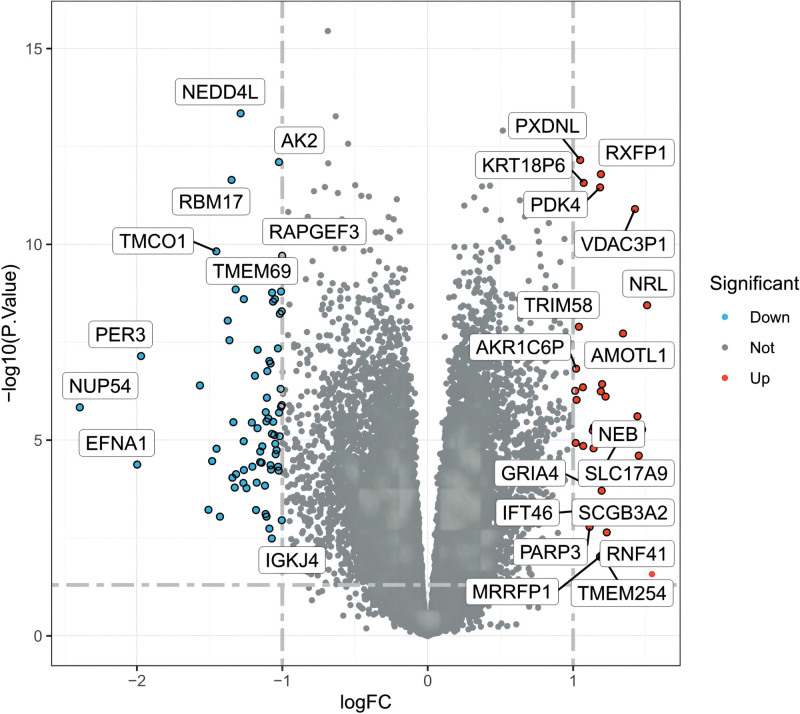
DEGs volcano plot. DEG = differentially expressed gene.

We screened a total of 24,544 DEGs in the controlled and uncontrolled samples. Compared to the controlled samples, we screened the data according to the conditions and eventually obtained 104 genes. Among them, 72 genes were downregulated and 32 genes were upregulated in the uncontrolled samples. A heat map and volcano map of the DEGs are shown in Figures 2 and 3, respectively.

### 3.2. WGCNA and co-expressed genes

**Figure 4.**
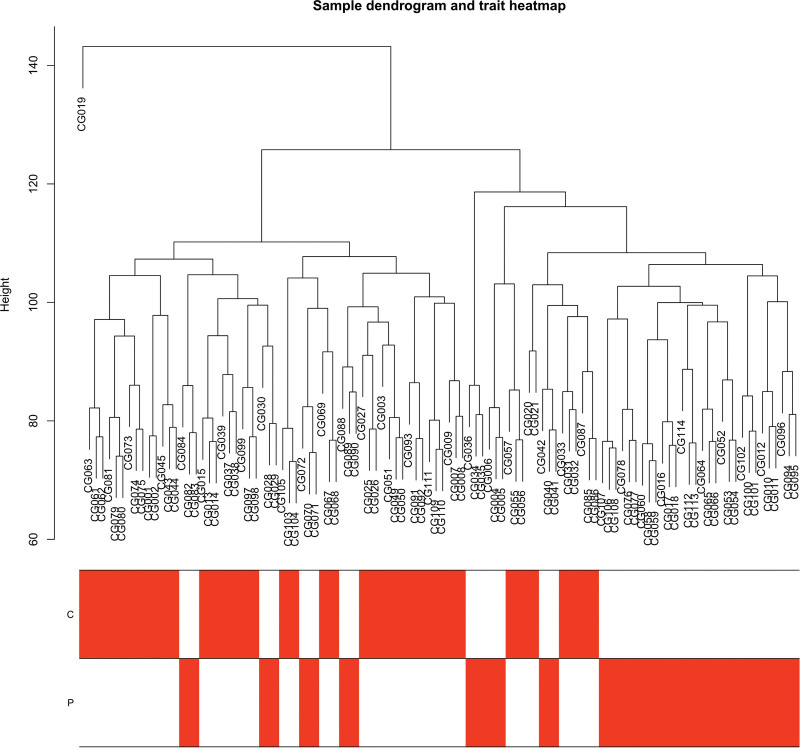
Sample dendrogram and trait heatmap.

**Figure 5.**
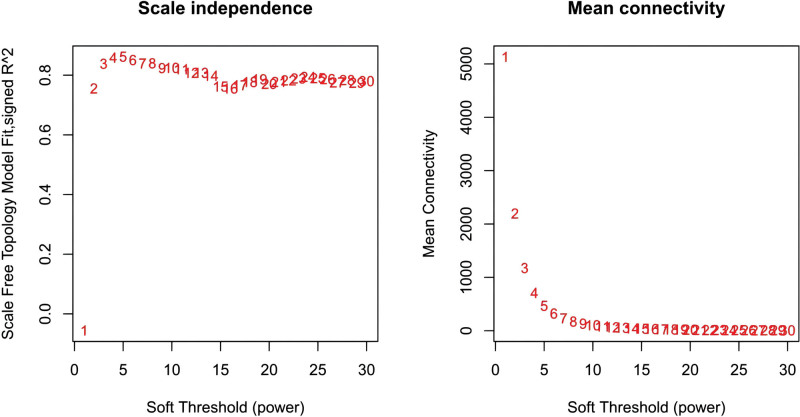
Scale independence.

**Figure 6.**
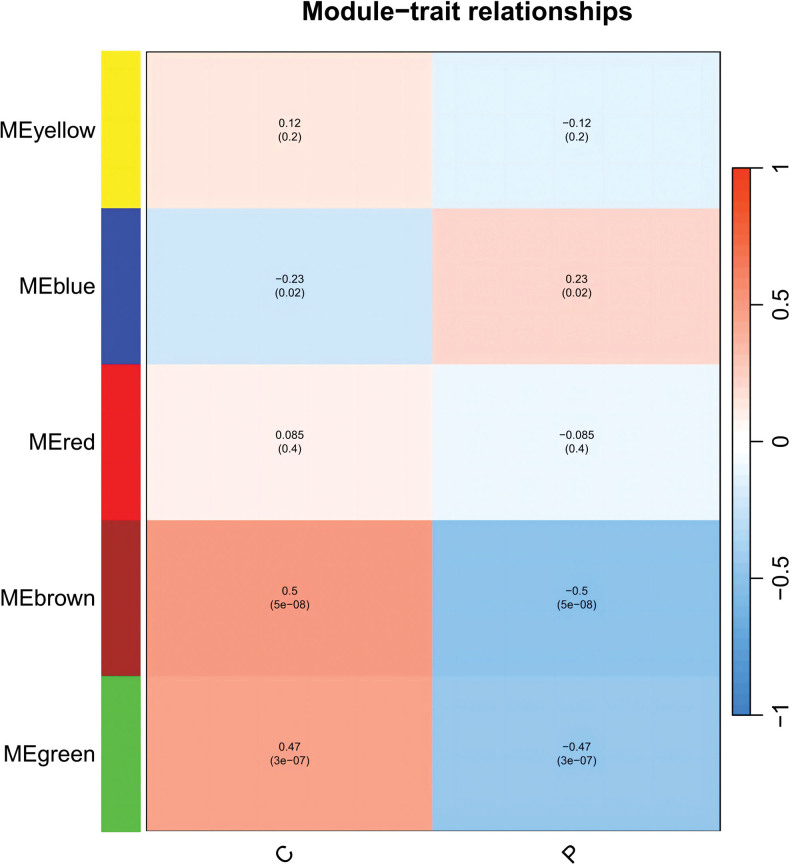
Module-trait relationships.

**Figure 7.**
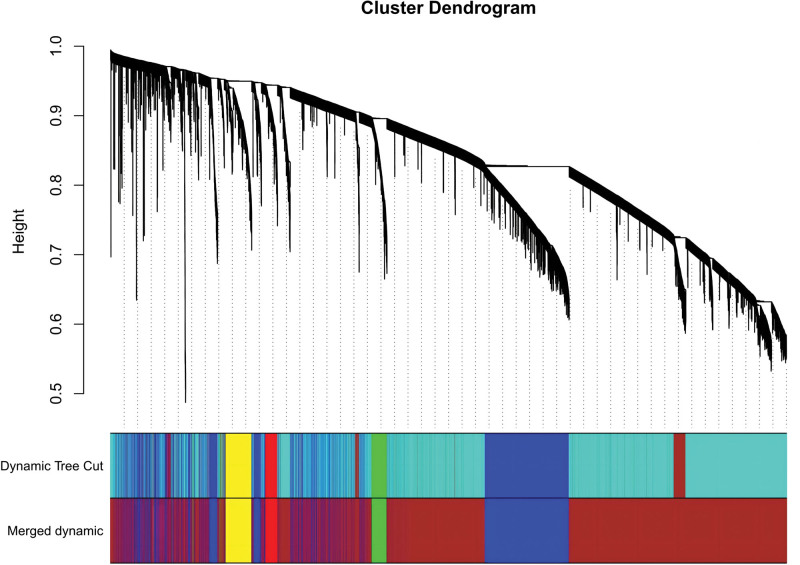
merged dynamic.

**Figure 8.**
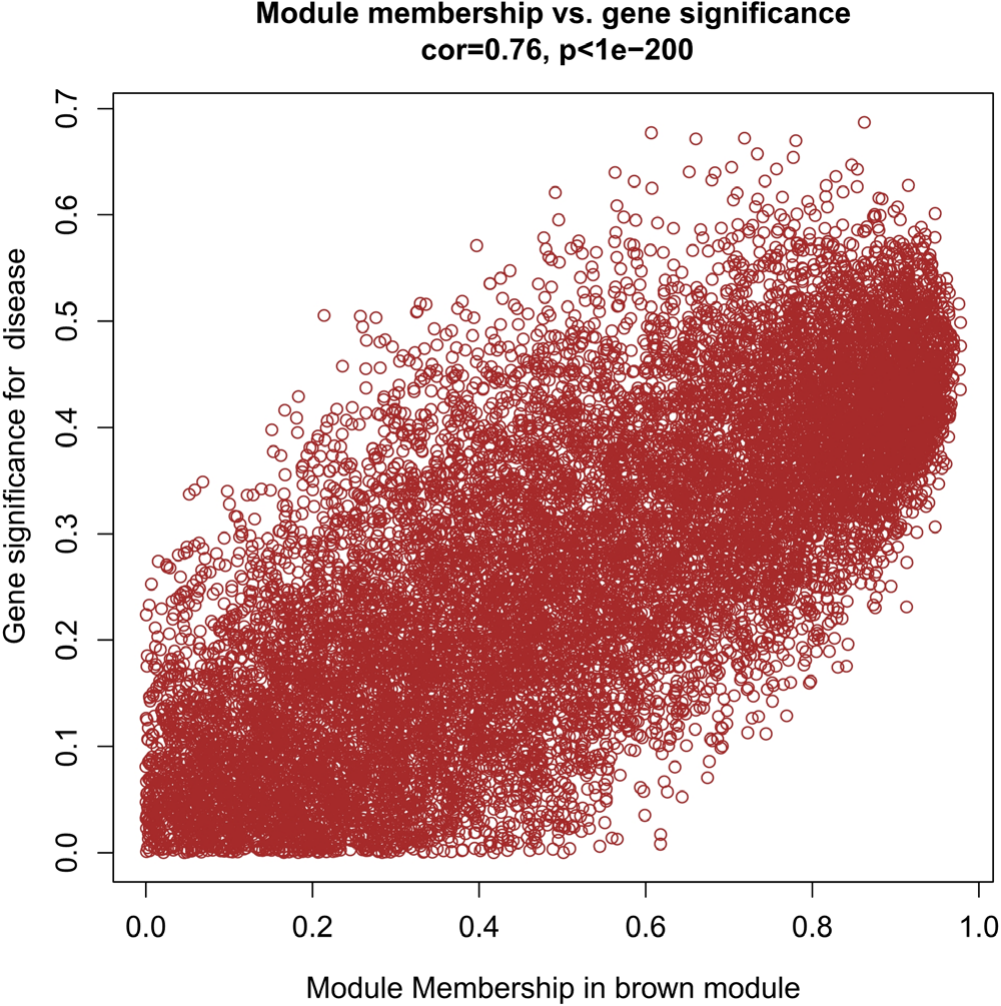
Module membership in brown module.

**Figure 9.**
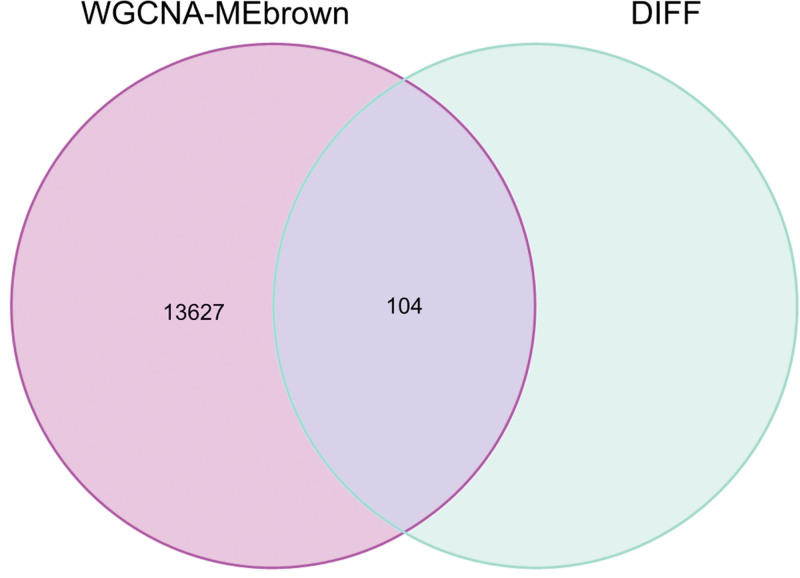
co-expressed genes.

In this study, we conducted a WGCNA to screen the DEGs associated with the disease. We constructed a sample clustering tree (Fig. 4), set a soft threshold of β = 5 (Fig. 5), used the dynamic clipping tree method to initially identify the modules, merged the similar modules, set the minimum number of genes for each gene network module to 30, and ultimately obtained 5 modules, of which the gray modules could not be aggregated with the other modules. (Fig. 6), Based on the module-trait associations (Fig. 7), the MEbrown module was picked as a key module. (*R* = 0.5, *P* = 5e-08). 13,731 genes were screened in the brown module (Fig. 8). We intersected the DEGs and genes from the MEbrown module identified using WGCNA and obtained 104 co-expressed genes. (Fig. 9).

### 3.3. Enrichment analysis

**Figure 10.**
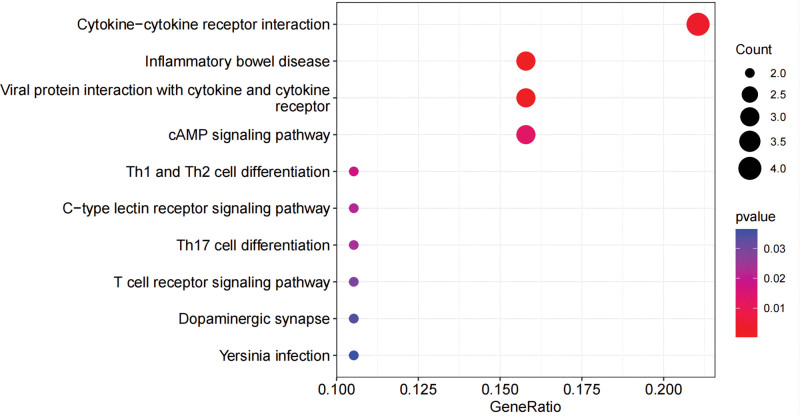
KEGG. KEGG = Kyoto encyclopedia of genes and genomes.

**Figure 11.**
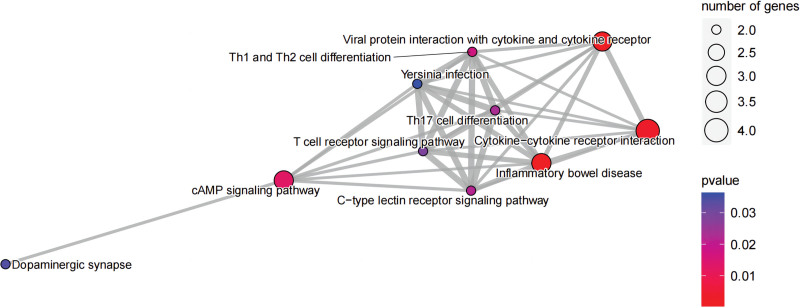
KEGG net. KEGG = Kyoto encyclopedia of genes and genomes.

**Figure 12.**
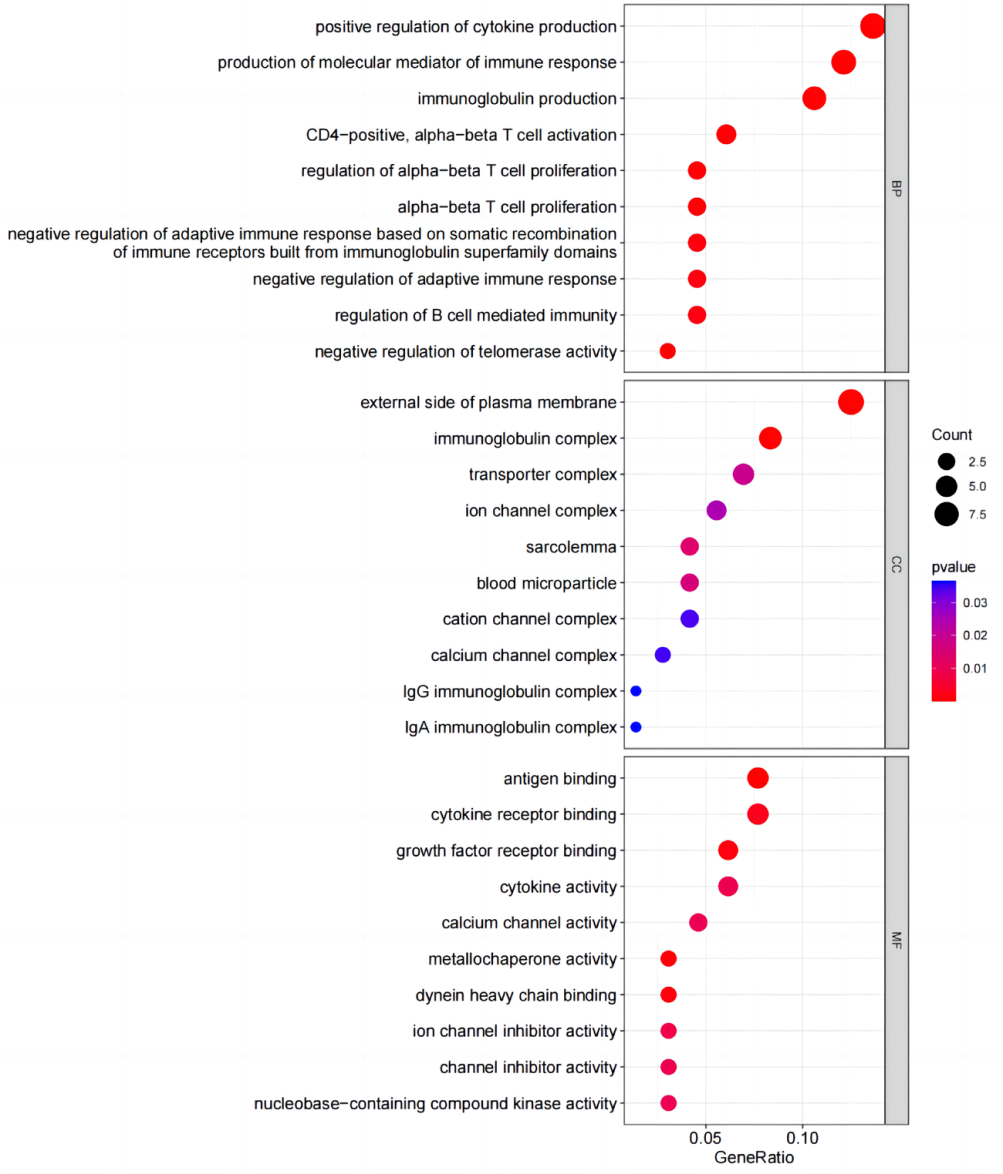
GO. GO = gene ontology.

The functional analysis of co-expressed genes identified the related pathways of pediatric asthma and analyzed the possible roles of these genes in the related pathways. After integrating relevant data sources, the pathways were mapped as shown in KEGG and GO. In KEGG, these genes are closely related to inflammatory bowel disease, the cAMP signaling pathway, viral protein interaction with cytokine and cytokine receptor, Th1 and Th2 cell differentiation, and cytokine-cytokine receptor interaction (Fig. 10, Table 1). In addition, there are some connections between these pathways or functions (Fig. 11). GO analysis includes biological process, cellular component, and molecular function (Fig. 12, Table 2). Biological process mainly includes positive regulation of cytokine production, negative regulation of telomerase activity, regulation of B cell-mediated immunity, negative regulation of adaptive immune response, and negative regulation of adaptive immune response based on somatic recombination of immune receptors built from immunoglobulin superfamily domains. Cellular component mainly includes the external side of the plasma membrane, sarcolemma, and multiple complexes. Such as immunoglobulin complex, transporter complex, IgG/IgA immunoglobulin complex, and so on. Molecular function also includes multiple bindings and activities. For example, antigen binding, cytokine receptor binding, cytokine activity, and calcium channel activity.

**Table 1:** KEGG enrichment analyses.

ID	Description	*P* value
hsa05321	Inflammatory bowel disease	<.001
hsa04061	Viral protein interaction with cytokine and cytokine receptor	.001
hsa04060	Cytokine-cytokine receptor interaction	.004
hsa04024	cAMP signaling pathway	.013
hsa04658	Th1 and Th2 cell differentiation	.017
hsa04625	C-type lectin receptor signaling pathway	.022
hsa04659	Th17 cell differentiation	.023
hsa04660	T cell receptor signaling pathway	.029
hsa04728	Dopaminergic synapse	.034
hsa05135	Yersinia infection	.036

KEGG = Kyoto encyclopedia of genes and genomes.

**Table 2:** GO enrichment analyses.

Ontology	ID	Description	*P* value
BP	GO: 0002377	Immunoglobulin production	<.001
BP	GO: 0002440	Production of molecular mediator of immune response	<.001
BP	GO: 0001819	Positive regulation of cytokine production	<.001
BP	GO: 0046640	Regulation of alpha-beta T cell proliferation	<.001
BP	GO: 0051974	Negative regulation of telomerase activity	<.001
BP	GO: 0046633	Alpha-beta T cell proliferation	<.001
BP	GO: 0035710	CD4-positive, alpha-beta T cell activation	<.001
BP	GO: 0002823	Negative regulation of adaptive immune response based on somatic recombination of immune receptors built from immunoglobulin superfamily domains	.001
BP	GO: 0002820	Negative regulation of adaptive immune response	.001
BP	GO: 0002712	Regulation of B cell-mediated immunity	.001
CC	GO: 0019814	Immunoglobulin complex	<.001
CC	GO: 0009897	External side of plasma membrane	<.001
CC	GO: 0042383	Sarcolemma	.014
CC	GO: 0072562	Blood microparticle	.016
CC	GO: 1990351	Transporter complex	.019
CC	GO: 0034702	Ion channel complex	.025
CC	GO: 0034703	Cation channel complex	.034
CC	GO: 0034704	Calcium channel complex	.034
CC	GO: 0071735	IgG immunoglobulin complex	.036
CC	GO: 0071745	IgA immunoglobulin complex	.036
MF	GO: 0003823	Antigen binding	<.001
MF	GO: 0016530	Metallochaperone activity	.001
MF	GO: 0045504	Dynein heavy chain binding	.001
MF	GO: 0070851	Growth factor receptor binding	.001
MF	GO: 0005126	Cytokine receptor binding	.003
MF	GO: 0008200	Ion channel inhibitor activity	.009
MF	GO: 0016248	Channel inhibitor activity	.009
MF	GO: 0005125	Cytokine activity	.010
MF	GO: 0005262	Calcium channel activity	.010
MF	GO: 0019205	Nucleobase-containing compound kinase activity	.010

BP = biological process, CC = cellular component, GO = gene ontology, MF = molecular function.

### 3.4. Identification of hub genes

**Figure 13.**
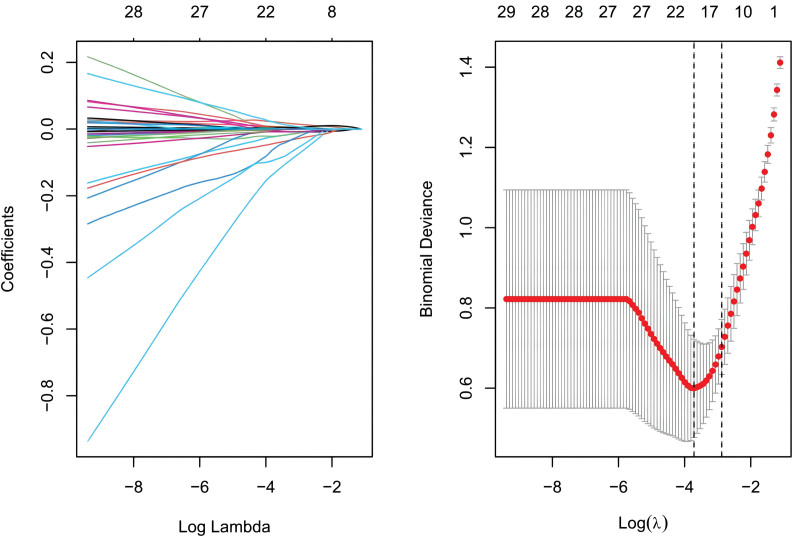
LASSO. LASSO = lasso regression algorithm.

**Figure 14.**
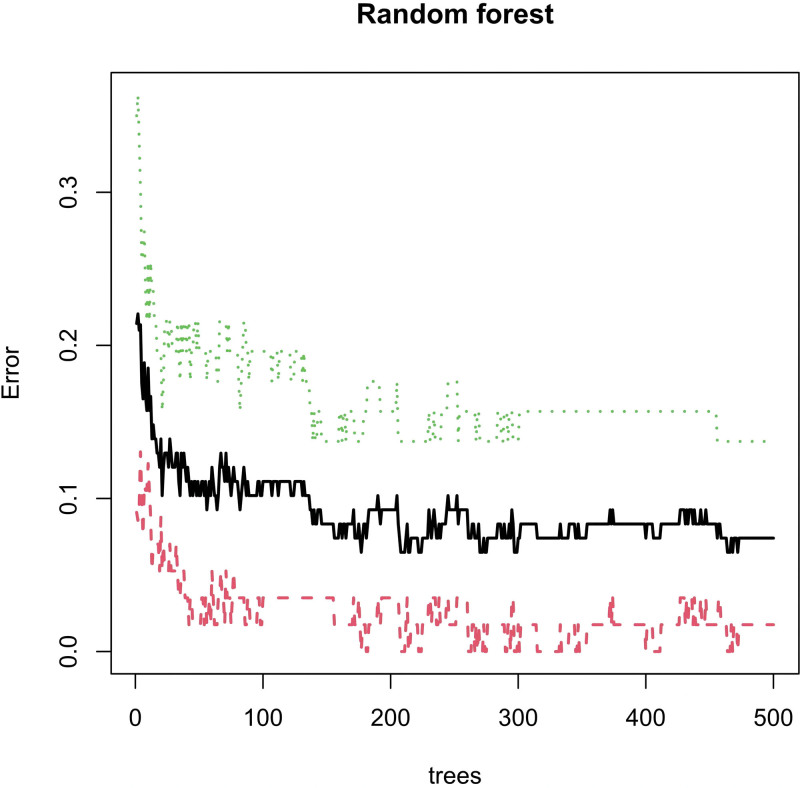
Random forest.

**Figure 15.**
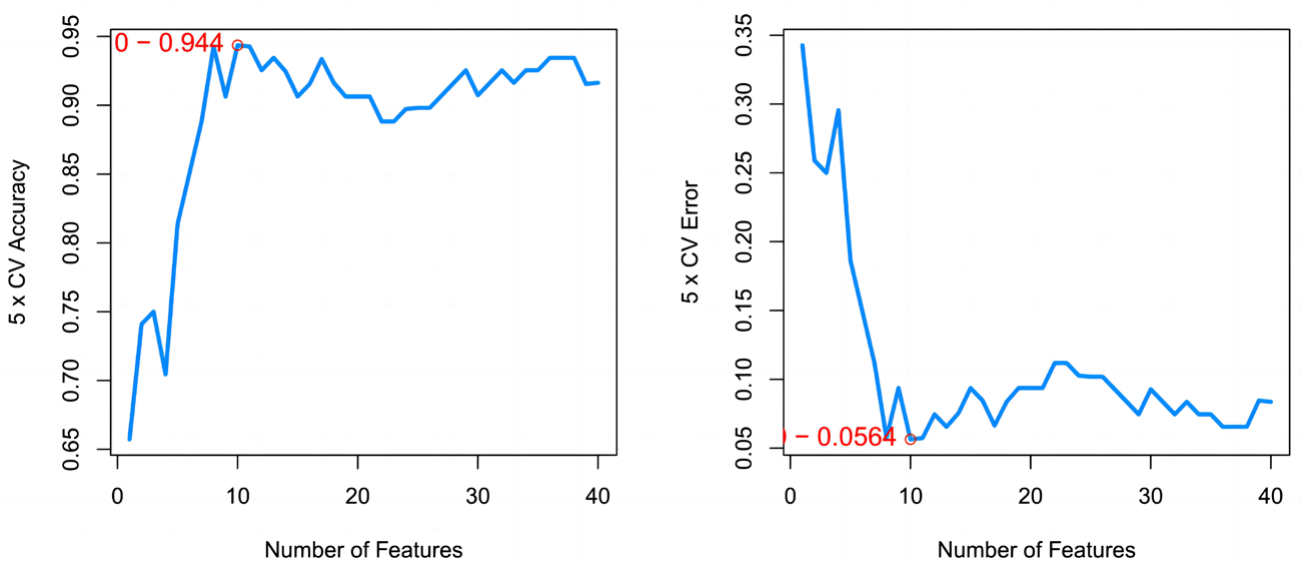
SVM-REF. SVM-REF = support vector machine-recursive feature elimination.

**Figure 16.**
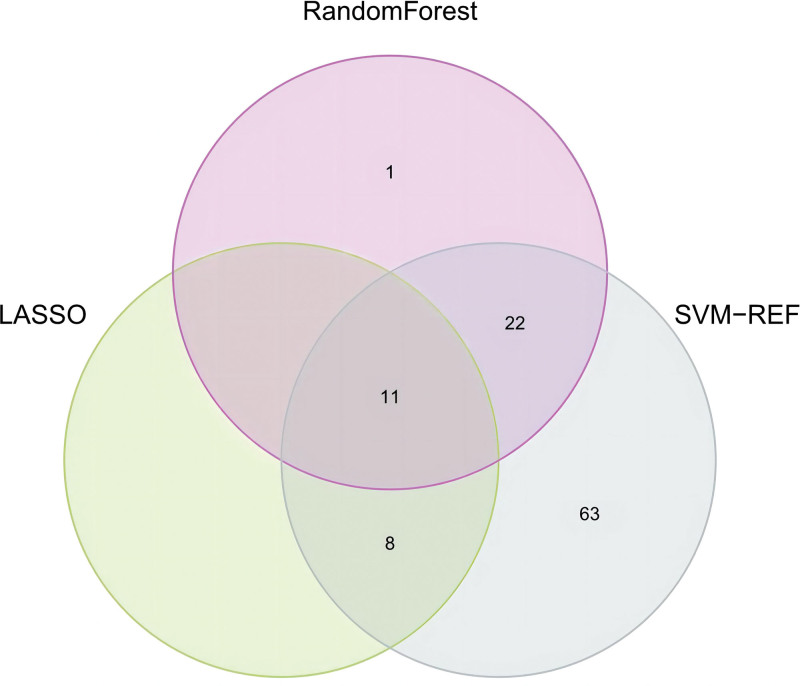
Hub genes.

We conducted Random Forest, LASSO, and support vector machine-recursive feature elimination (SVM-REF) screening in order to further identify the hub genes. Through LASSO screening, we obtained 19 genes (Fig. 13). Through Random Forest screening, we obtained 34 genes (Fig. 14). Through SVM-REF screening, we obtained 104 genes (Fig. 15). After the intersection of the genes screened using Random Forest, LASSO, and SVM-REF, 11 hub genes were obtained. They were AK2, PDK4, PER3, GZMH, NUMBL, NRL, SCO2, CREBZF, LARP1B, RXFP1, and VDAC3P1 (Fig. 16).

### 3.5. Verification of hub genes

**Figure 17.**
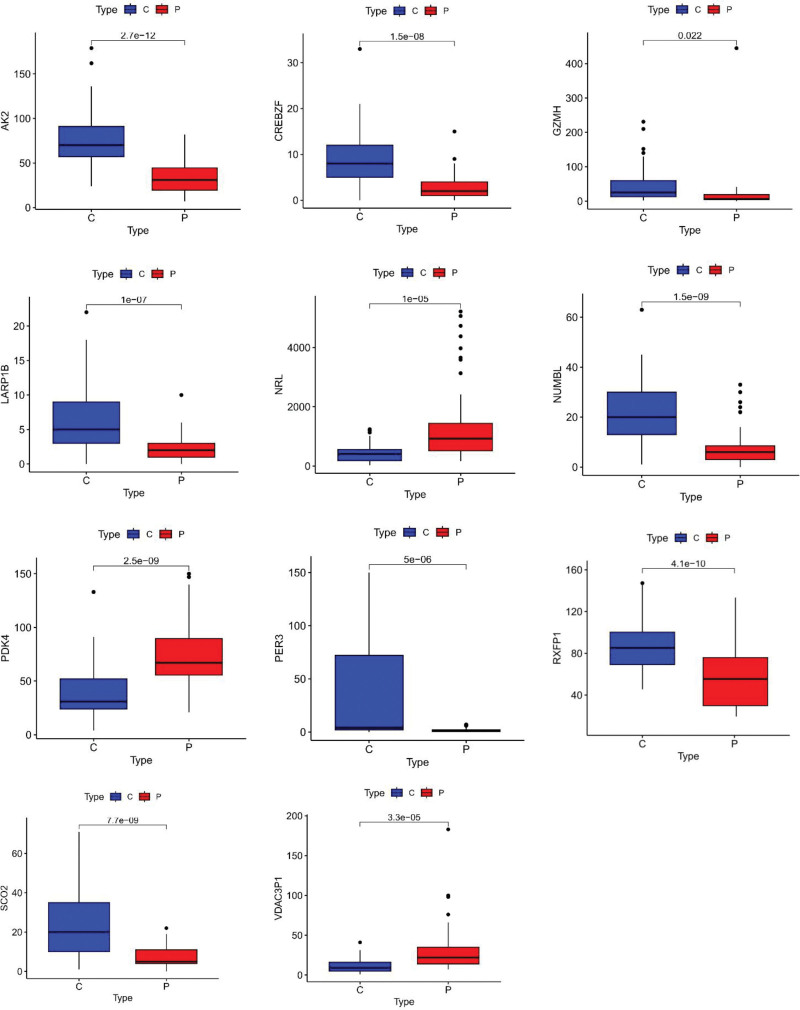
Box plots of hub genes.

**Figure 18.**
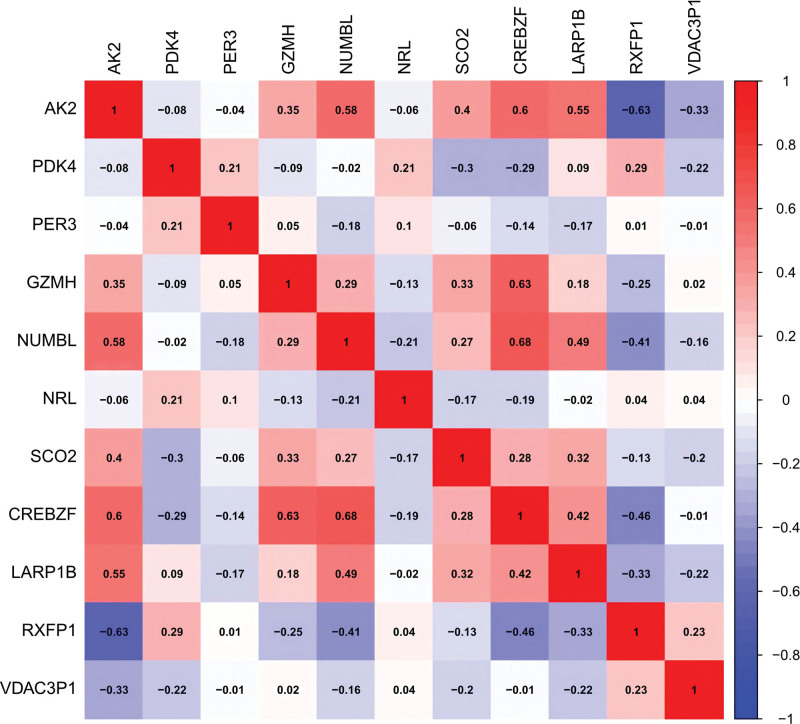
heatmap analysis of hub genes.

We further evaluated the diagnostic efficacy of these 11 genes using ROC curves in the GSE135192 dataset. As exhibited in Table 3, these genes were associated with high levels of accuracy. The AUC was > 0.8 for more than half the genes. As for the remaining genes, their AUC was more than 0.78. We thought that they were good. Meanwhile, we assessed the expression profiles of the 11 genes. These genes displayed significantly differential expression between the control and patient groups (Fig. 17). We also performed a correlation heatmap analysis for hub genes (Fig. 18). Some genes are positively correlated (e.g., CREBZF and GZMH), while some genes are negatively correlated (e.g., AK2 and RXFP1).

**Table 3:** AUC of hub genes.

Gene	AUC
AK2	0.873
CREBZF	0.814
GZMH	0.796
LARP1B	0.783
NRL	0.792
NUMBL	0.835
PDK4	0.834
PER3	0.796
RXFP1	0.832
SCO2	0.827
VDAC3P1	0.819

AUC = area under curve.

### 3.6. ssGSEA

**Figure 19.**
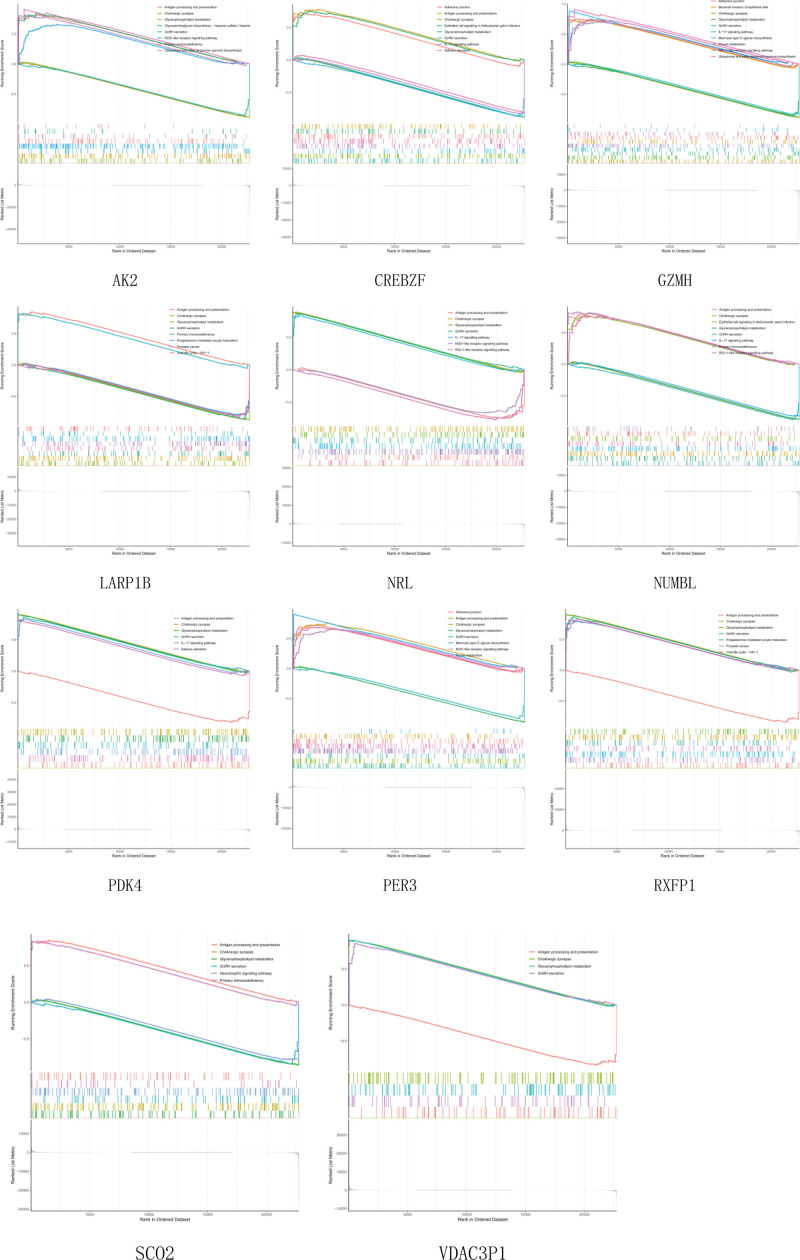
ssGSEA. ssGSEA = single sample gene set enrichment analysis.

**Figure 20.**
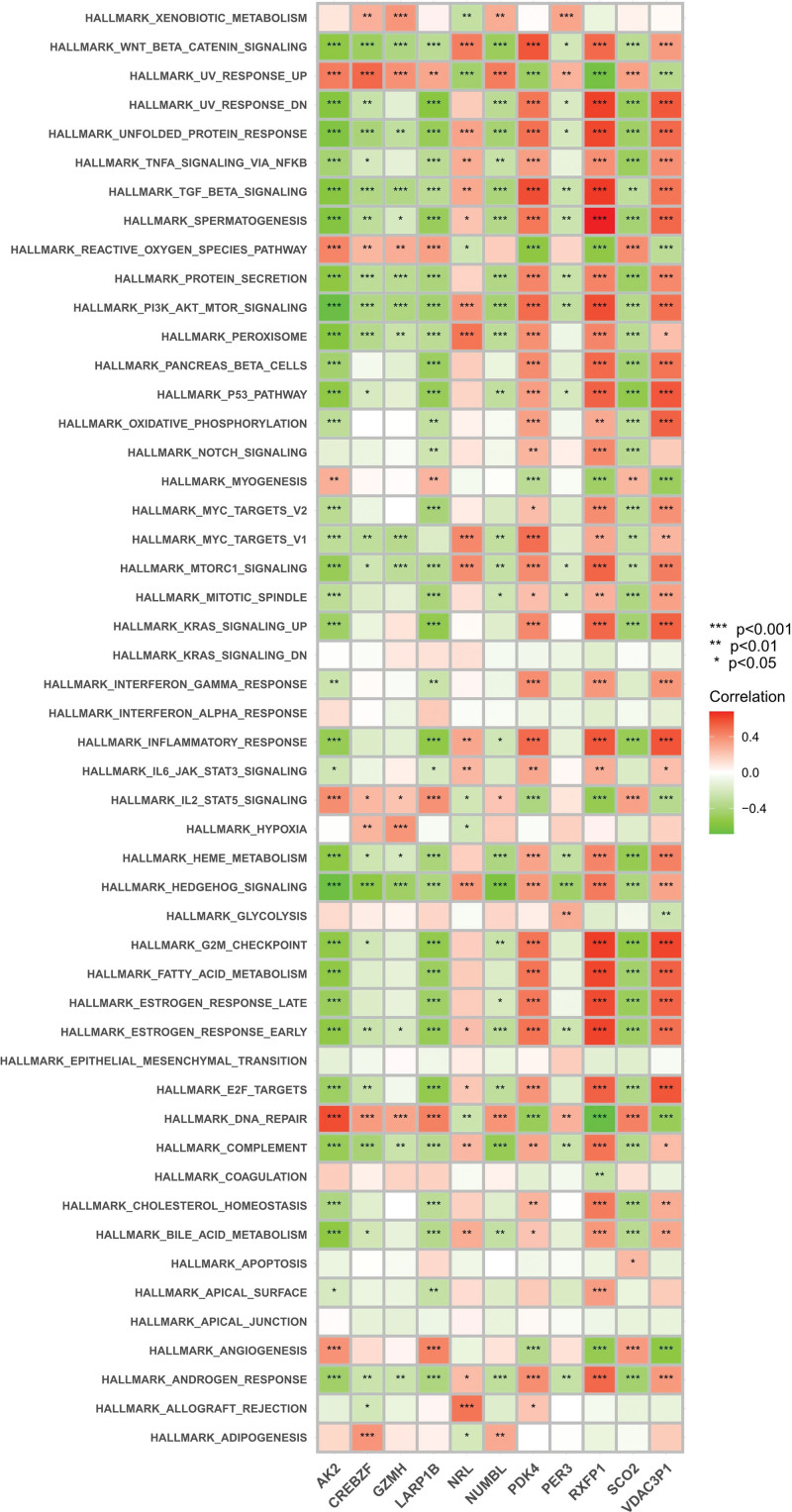
Immunity heat map.

To further understand how these genes function, we performed ssGSEA analysis (Fig. 19). The ssGSEA principle is similar to GSEA, except that GSEA requires a gct format expression matrix, and then the genes are ranked according to the differences in gene expression between samples to obtain rank values for subsequent analysis. While rank ranking values for genes could not be calculated by differences in a single sample, ssGSEA was used as rank values for that gene by ranking all gene expression in the sample.^[10]^ In GSEA analysis, these genes were highly functionally similar and were all related to pathways and functions such as the NOD-like receptor signaling pathway, the IL-17 signaling pathway, the RIG-I-like receptor signaling pathway, neurotrophin signaling pathway, and cholinergic synapse. The correlation of these single genes was tested by functional analysis of marker gene sets (Fig. 20). AK2, LARP1B, and SCO2 showed negative correlations with most functions and pathways, while PDK4, RXFP1, and VDAC3P1 showed positive correlations, and most of them were significant. It may not be so accurate, but to some extent, this can be verified against Figure 18.

## 4. Disscuss

RXFP1 is responsible for encoding relaxin receptor 1, a G protein-coupled receptor that is widespread in multiple organs such as the heart, blood vessels, kidneys, and lungs.^[11]^ The main function of relaxin receptor 1 is to regulate the biological activity of relaxin, such as lowering blood pressure, resisting inflammation, and preventing fibrosis.^[12]^ The role of RXFP1 may be complex during asthma pathology. Experimental studies have revealed that chronic relaxin treatment in animal models of airway disease can have positive effects, such as inhibiting airway hyperresponsiveness and reversing established fibrosis, and these results imply its potential therapeutic effects.^[13]^ In addition, relaxin has been found to be able to counteract acute contraction by reducing bronchoconstrictors released from mast cells, as well as directly inducing bronchodilation, chronic relaxin treatment in animal models of airway disease can have positive effects, such as inhibiting airway hyperresponsiveness and reversing established fibrosis, and these results imply its potential therapeutic effects.^[14]^ The expression of RXFP1 may be decreased in the respiratory epithelial cells of asthmatic patients, which may be associated with respiratory inflammation and remodeling. First, a decrease in RXFP1 gene expression may weaken the anti-inflammatory effects of relaxin.^[15]^ Relaxin is able to inhibit the activity of a variety of inflammatory cells, such as eosinophils, lymphocytes, and mast cells, which play a key role in the inflammatory response to asthma. Second, a decrease in RXFP1 gene expression may weaken the anti-fibrotic effect of relaxin.^[16]^ Thus, decreased RXFP1 gene expression may exacerbate airway inflammation and remodeling, thereby worsening asthma symptoms. However, regarding the specific role of RXFP1 in asthma, existing studies are not deep enough, and further experiments are needed to explore. For example, RXFP1 can be knocked out in animal models by gene editing technology to observe its effect on the pathological process of asthma. In addition, the effect of RXFP1 gene expression on asthma symptoms can also be observed through pharmacological intervention. In summary, RXFP1 may play a key role in the pathogenesis of asthma, but the specific mechanism needs to be clarified by further studies. Understanding the role of RXFP1 in asthma may help to develop new therapeutic strategies and improve the quality of life of patients with asthma.

AK2 encodes adenosine kinase 2, an enzyme that plays an important role in cells and is mainly involved in cellular energy metabolism and signal transduction.^[17]^ At present, the direct link between AK2 and asthma has not been clarified. However, we can make some possible inferences. For example, mutations in AK2 may affect energy conversion and signal transmission in cells, which may have an impact on the function of the immune system. Abnormal immune system response is one of the main factors causing asthma, so if AK2 mutations lead to immune system dysfunction, then this may increase the risk of asthma in patients. In addition, variants in AK2 may also affect cell growth and differentiation,^[18]^ which may have an impact on the health of the respiratory tract. If mutations in AK2 lead to abnormal growth and differentiation of airway epithelial cells, then this may trigger an inflammatory response in the airways, thereby increasing the risk of asthma.^[19]^

PDK4 is an enzyme that plays an important role in cells, and it plays a key role in regulating the processes of cells,^[20]^ especially in glucose metabolism and fatty acid oxidation. These processes are essential for energy production and use in cells, and PDK4 plays an important role in maintaining the normal function of cells. Cells of the immune system, such as T cells and B cells, require a lot of energy to perform their functions, including proliferation, differentiation, and the generation of immune responses. If the function of PDK4 is affected, it may alter the energy metabolism of cells and thus affect the function of immune cells.^[21]^ The role of PDK4 in airway inflammation or lung function is not clear; however, this does not mean that PDK4 is completely unrelated to asthma because the pathogenesis of asthma is multifactorial, including genetic, environmental, and lifestyle factors. We can speculate that PDK4 may affect the immune system by affecting cellular energy metabolism, which leads to the development of asthma. Future studies may shed light on the potential role of PDK4 in asthma.

Compared with other articles on pediatric asthma markers, our study employed multiple analytical methods, including WGCNA, LASSO, Random Forest, and SVM-RFF, to screen for genes associated with childhood asthma. These methods can help us find key genes involved in disease initiation and progression. Our study also found that the pathogenesis of childhood asthma is closely related to multiple factors such as immune, neurological, psychiatric, endocrine, and genetic factors, as well as abnormal signaling pathways. This brings some difficulties for clinical treatment, but it also provides important clues for the study of molecular mechanisms.

There are inevitable limitations to this study. First of all, it is limited objectively, and the results cannot be verified well by experiments. Second, based on the results obtained from a certain dataset, it may not be very accurately applied to the entire disease.

## 5. Conclusion

Our study is important for understanding the pathogenesis of childhood asthma and developing new diagnostic and therapeutic approaches. It is hoped that our findings may contribute to improving the quality of life of pediatric asthma patients.

## Author contributions

**Conceptualization:** Yijie Wang, Yongjun Li.

**Methodology:** Yijie Wang.

**Supervision:** Youpeng Wang.

**Validation:** Youpeng Wang.

**Writing – original draft:** Kexin Lin, Youpeng Wang.

**Writing – review & editing:** Kexin Lin.
